# An LaeA- and BrlA-Dependent Cellular Network Governs Tissue-Specific Secondary Metabolism in the Human Pathogen *Aspergillus fumigatus*

**DOI:** 10.1128/mSphere.00050-18

**Published:** 2018-03-14

**Authors:** Abigail L. Lind, Fang Yun Lim, Alexandra A. Soukup, Nancy P. Keller, Antonis Rokas

**Affiliations:** a Department of Biomedical Informatics, Vanderbilt University School of Medicine, Nashville, Tennessee, USA; b Department of Medical Microbiology & Immunology, University of Wisconsin—Madison, Madison, Wisconsin, USA; c Department of Genetics, University of Wisconsin—Madison, Madison, Wisconsin, USA; d Department of Biological Sciences, Vanderbilt University, Nashville, Tennessee, USA; Carnegie Mellon University

**Keywords:** biosynthetic gene cluster, conidia, hyphal growth, hypoxia, mycelial growth, specialized metabolism, *srbA*, velvet protein complex

## Abstract

Biosynthesis of many ecologically important secondary metabolites (SMs) in filamentous fungi is controlled by several global transcriptional regulators, like the chromatin modifier LaeA, and tied to both development and vegetative growth. In *Aspergillus* molds, asexual development is regulated by the BrlA > AbaA > WetA transcriptional cascade. To elucidate BrlA pathway involvement in SM regulation, we examined the transcriptional and metabolic profiles of Δ*brlA*, Δ*abaA*, and Δ*wetA* mutant and wild-type strains of the human pathogen *Aspergillus fumigatus*. We find that BrlA, in addition to regulating production of developmental SMs, regulates vegetative SMs and the SrbA-regulated hypoxia stress response in a concordant fashion to LaeA. We further show that the transcriptional and metabolic equivalence of the Δ*brlA* and Δ*laeA* mutations is mediated by an LaeA requirement preventing heterochromatic marks in the *brlA* promoter. These results provide a framework for the cellular network regulating not only fungal SMs but diverse cellular processes linked to virulence of this pathogen.

**IMPORTANCE** Filamentous fungi produce a spectacular variety of small molecules, commonly known as secondary or specialized metabolites (SMs), which are critical to their ecologies and lifestyles (e.g., penicillin, cyclosporine, and aflatoxin). Elucidation of the regulatory network that governs SM production is a major question of both fundamental and applied research relevance. To shed light on the relationship between regulation of development and regulation of secondary metabolism in filamentous fungi, we performed global transcriptomic and metabolomic analyses on mutant and wild-type strains of the human pathogen *Aspergillus fumigatus* under conditions previously shown to induce the production of both vegetative growth-specific and asexual development-specific SMs. We find that the gene *brlA*, previously known as a master regulator of asexual development, is also a master regulator of secondary metabolism and other cellular processes. We further show that *brlA* regulation of SM is mediated by *laeA*, one of the master regulators of SM, providing a framework for the cellular network regulating not only fungal SMs but diverse cellular processes linked to virulence of this pathogen.

## Abstract (precis)

Filamentous fungi produce a spectacular variety of small molecules, commonly known as secondary or specialized metabolites (SMs), which are critical to their ecologies and lifestyles (e.g., penicillin, cyclosporine, and aflatoxin). Elucidation of the regulatory network that governs SM production is a major question of both fundamental and applied research relevance. To shed light on the relationship between regulation of development and regulation of secondary metabolism in filamentous fungi, we performed global transcriptomic and metabolomic analyses on mutant and wild-type strains of the human pathogen *Aspergillus fumigatus* under conditions previously shown to induce the production of both vegetative growth-specific and asexual development-specific SMs. We find that the gene *brlA*, previously known as a master regulator of asexual development, is also a master regulator of secondary metabolism and other cellular processes. We further show that *brlA* regulation of SM is mediated by *laeA*, one of the master regulators of SM, providing a framework for the cellular network regulating not only fungal SMs but diverse cellular processes linked to virulence of this pathogen.

## INTRODUCTION

Filamentous fungi produce a remarkable diversity of specialized secondary metabolites (SMs), which are small molecules that play diverse ecological roles in fungal defense, communication, and virulence (1). In fungi, SMs are typically produced by pathways organized into contiguous biosynthetic gene clusters (BGCs), an organization atypical of metabolic pathways in most other eukaryotes (2). The transcription of these BGCs is often controlled by both cluster-specific transcription factors, as well as globally acting transcriptional regulators. These global regulators respond to a variety of environmental signals, including pH, temperature, light, and nutrient sources, to transcriptionally regulate BGCs and are typically well conserved in filamentous fungi (3).

Many of the environmental signals that regulate SM production in *Aspergillus* fungi, including temperature, pH, and carbon or nitrogen sources, also trigger the onset of asexual and sexual development (4). At the cellular level, this coupling between SM production and development is orchestrated in part by the velvet protein complex, which is composed of two velvet domain proteins, VelA and VelB, and the methyltransferase LaeA (5). Although the precise mechanism by which the velvet complex regulates the two processes is unknown, LaeA regulates transcription epigenetically through heterochromatin reorganization of target DNA (6, 7). The result of this coupling of SM and development is that several SMs show tissue specificity: i.e., they are localized or produced only in certain tissues. For example, the SMs 1,8-dihydroxynaphthalene-melanin (DHN-melanin), fumigaclavines, endocrocin, trypacidin, and fumiquinazolines appear to be localized to the asexual spores of *Aspergillus fumigatus* (8–12). Importantly, several of these asexual spore (conidial) metabolites, all LaeA regulated (13), are part of the pathogenic arsenal of this human pathogen (reviewed in reference 14).

In *Aspergillus*, asexual development is controlled by three regulatory genes sequentially expressed at specific stages of asexual fruiting body development (conidiation) (14). The first protein of this regulatory cascade, BrlA, accumulates in vegetative cells shortly before asexual development (15). In the middle stages of conidiation, BrlA activates AbaA, which controls the development of the asexual fruiting body (conidiophore) and spores (conidia). In late stages of asexual development, AbaA activates WetA, which is required for conidial maturation through governance of critical conidial cell wall components (15). Recent evidence suggests that these three regulators may also be involved in regulating the expression of BGCs whose SM products are specifically found in asexual spores (8, 12, 16, 17).

To shed light on the relationship between regulation of asexual development and tissue-specific regulation of secondary metabolism in filamentous fungi, we performed global transcriptomic and metabolomic analyses on Δ*brlA*, Δ*abaA*, and Δ*wetA* mutant and wild-type (WT) strains of *A. fumigatus* under conditions previously shown to induce the production of both vegetative growth-specific and asexual development-specific SMs (13). Our results show that BrlA positively regulates the transcriptional activity of 13 BGCs and their SMs; importantly, BrlA regulates not only the production of both asexual development-specific SMs and vegetative growth-specific SMs, but also the activity of several transcriptional regulators of diverse cellular processes, such as the SrbA-regulated hypoxia stress response. Remarkably, comparison of BrlA- and LaeA-regulated BGCs shows that nine BGCs (DHN-melanin, fumigaclavine, endocrocin, trypacidin, helvolic acid, fumisoquin, gliotoxin, fumiquinazoline, and pyripyropene A) appear to be identically regulated by both proteins. To further dissect this regulatory overlap between LaeA and BrlA, we used chromatin immunoprecipitation-quantitative PCR (ChIP-qPCR) to show that *laeA* loss results in heterochromatic marks in the *brlA* promoter and hence dampening of *brlA* expression; this finding is not only consistent with work showing that LaeA governs BrlA expression in *Penicillium oxalicum* (18) but also suggests that LaeA control of BrlA expression is evolutionarily conserved. The effect of LaeA activity on *brlA* transcript levels explains, to a large degree, the concordance of BGC regulation and hypoxia gene regulation by these two proteins. These results argue that LaeA and BrlA are key conserved components of the cellular network governing tissue-specific secondary metabolism as well as diverse cellular processes in filamentous fungi.

## RESULTS AND DISCUSSION

### Genome-wide transcriptional impact of BrlA, AbaA, and WetA.

To examine the genome-wide regulatory roles of the three central regulators of asexual development, we performed RNA sequencing on *A. fumigatus* wild-type (WT) and Δ*brlA*, Δ*abaA*, and Δ*wetA* mutant strains grown on minimal medium under conditions known to induce the production of both vegetative growth-specific and asexual development-specific SMs. A total of 6,738 of the 9,784 genes in the genome of the *A. fumigatus* Af293 strain were differentially expressed in the Δ*brlA* mutant versus WT comparison (3,358 overexpressed and 3,380 underexpressed) (Table 1; see Table S1 in the supplemental material). Fewer genes were differentially expressed in the Δ*abaA* versus WT comparison (1,895 differentially expressed genes: 1,148 overexpressed and 747 underexpressed) and in the Δ*wetA* mutant versus WT comparison (2,158 differentially expressed genes: 1,192 overexpressed and 966 underexpressed).

10.1128/mSphere.00050-18.3 TABLE S1  Differential gene expression of all strains. Download TABLE S1, XLSX file, 1.1 MB. Copyright © 2018 Lind et al. 2018 Lind et al. This content is distributed under the terms of the Creative Commons Attribution 4.0 International license.

**TABLE 1:** Numbers of differentially expressed genes between mutant and wild-type strains in all RNA-seq comparisons

Expression level	No. of genes differentially expressed		
	Δ*brlA* mutant in GMM	Δ*abaA* mutant in GMM	Δ*wetA* mutant in GMM
Overexpressed	3,358	1,148	1,192
Underexpressed	3,380	747	966

**FIG 1:**
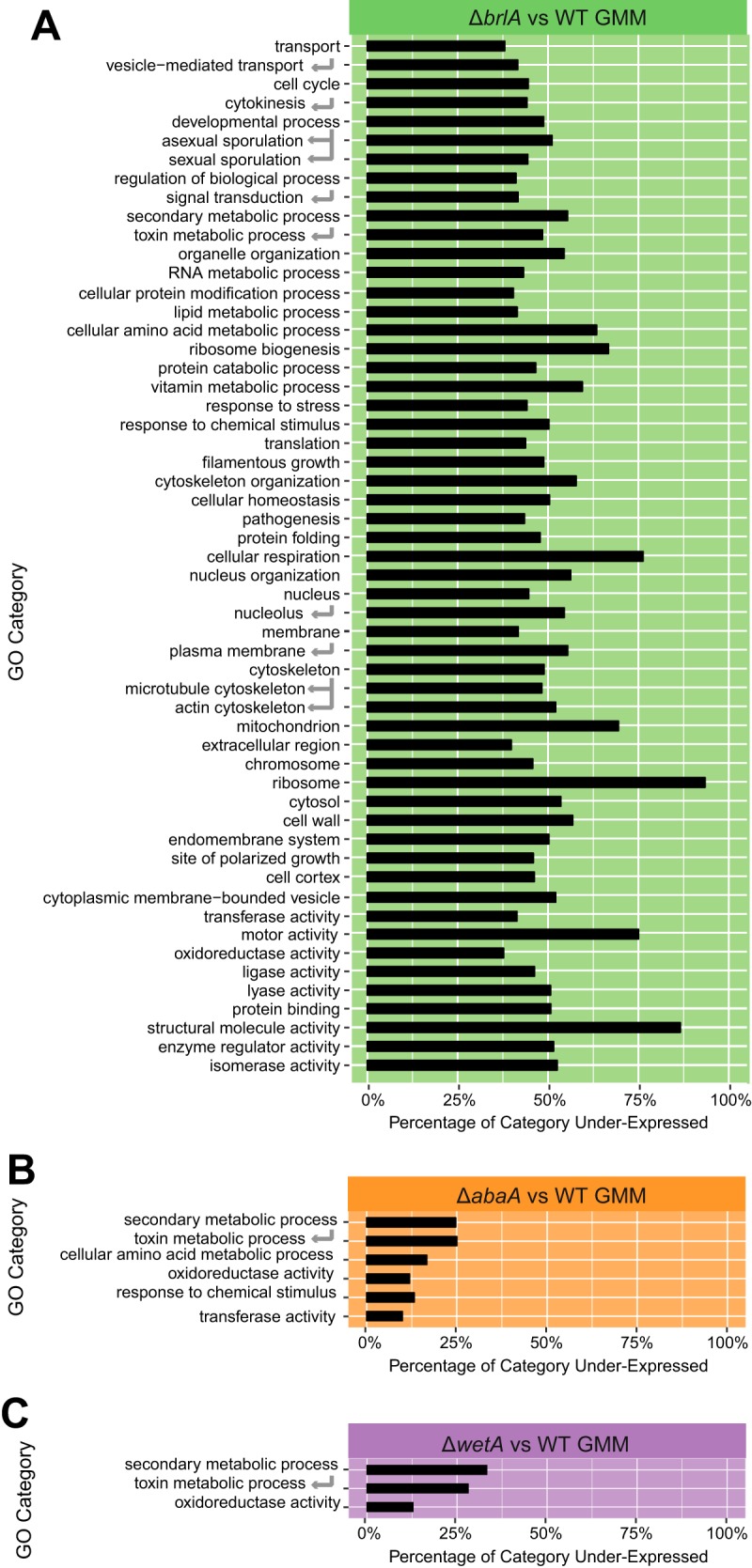
Genes underexpressed in the Δ*brlA* mutant are involved in a diverse set of cellular processes. Shown are results from Gene Ontology (GO) enrichment analysis for genes underexpressed in (A) Δ*brlA* (selected), (B) Δ*abaA*, and (C) Δ*wetA* mutant strains compared to the wild type. The percentage of underexpressed genes in each GO category was calculated by dividing the number of genes in the category that are underexpressed by the total number of genes in the category. Only a representative subset of categories is shown for the Δ*brlA* mutant; the full list of statistically enriched categories is provided in Table S2.

To determine processes positively regulated by BrlA, AbaA, and WetA, we performed Gene Ontology (GO) enrichment analysis for genes underexpressed in each deletion strain. Among the 55 functional categories enriched in genes underexpressed in the Δ*brlA* mutant versus the WT were secondary metabolism, response to stress, developmental process, asexual sporulation, cellular respiration, ribosome, mitochondrion, and structural molecule activity (Fig. 1A; see Table S2 in the supplemental material). Genes underexpressed in the Δ*abaA* mutant versus WT were enriched for six functional categories, namely, secondary metabolic process, oxidoreductase activity, cellular amino acid metabolic process, response to chemical stimulus, toxin metabolic process, and transferase activity (Fig. 1B; Table S2). Finally, genes underexpressed in the Δ*wetA* mutant versus WT were enriched for three categories, which were secondary metabolic process, toxin metabolic process, and oxidoreductase activity (Fig. 1C; Table S2).

10.1128/mSphere.00050-18.4 TABLE S2  All Gene Ontology (GO) enrichment results. Download TABLE S2, XLSX file, 0.1 MB. Copyright © 2018 Lind et al. 2018 Lind et al. This content is distributed under the terms of the Creative Commons Attribution 4.0 International license.

### BrlA is a key regulator of BGCs and SMs.

**FIG 2:**
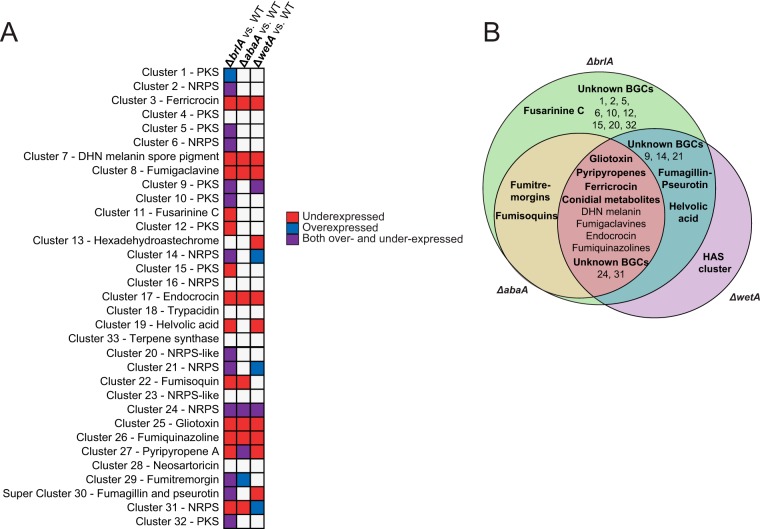
BrlA, AbaA, and WetA transcriptionally regulate many biosynthetic gene clusters (BGCs) involved in secondary metabolism. Shown is expression of all secondary metabolic BGCs in *Aspergillus fumigatus* in all strains tested. (A) Expression in the Δ*brlA*, Δ*abaA*, and Δ*wetA* mutants. BGCs in which half or more of the genes are overexpressed (overexpressed clusters) are shown in blue, BGCs in which half or more of the genes BGC are underexpressed (underexpressed clusters) are shown in red, and BGCs in which half or more of the genes were differentially expressed but did not have half or more genes either overexpressed or underexpressed (mixed expression) are shown in purple. (B) Overlap between BGCs underexpressed in the Δ*brlA*, Δ*abaA*, and Δ*wetA* mutants.

As BrlA is a key developmental regulator, we questioned whether its regulation of secondary metabolism is limited to metabolites associated with asexual spores. To answer this, we examined the transcriptional responses of the all 33 *A. fumigatus* BGCs (see Table S3 in the supplemental material) (19). While only five characterized BGCs (DHN-melanin, endocrocin, trypacidin, fumigaclavine, and fumiquinazoline) have been reported to be highly induced during asexual development, we found that 27/33 (82%) BGCs are differentially expressed in one or more of the three mutants examined (Fig. 2A), suggesting a much broader governance of SM production by these transcriptional regulators of asexual development. To confirm this trend, backbone synthesis genes from selected gene clusters were assayed by semiquantitative reverse transcription-PCR (RT-PCR) and showed the same trends observed from the transcriptome sequencing (RNA-seq) analysis (see Fig. S2 in the supplemental material). Like the trend observed with genome-wide transcriptional impact of these regulators, we find BrlA to be a major contributor to changes in BGC expression, regulating all but one of the differentially expressed BGCs (26/27 [96%]), followed by WetA (15/27 [45%]) and AbaA (11/27 [34%]) (Fig. 2A). Of the 27 differentially expressed BGCs, 9 were regulated by all three transcriptional regulators, 10 showed BrlA-specific regulation, 1 showed WetA-specific regulation, 2 showed joint regulation by both BrlA and AbaA, and 5 showed joint regulation by BrlA and WetA (Fig. 2B). These results suggest that, unlike BrlA, WetA (with the exception of one BGC) and AbaA do not independently regulate their BGC targets.

10.1128/mSphere.00050-18.5 TABLE S3  All secondary metabolic gene clusters in *Aspergillus fumigatus*. Download TABLE S3, XLSX file, 0.1 MB. Copyright © 2018 Lind et al. 2018 Lind et al. This content is distributed under the terms of the Creative Commons Attribution 4.0 International license.

10.1128/mSphere.00050-18.6 TABLE S4  *t* tests for significant differences between supernatant and mycelial secondary metabolites shown in Fig. S1. Download TABLE S4, XLSX file, 0.1 MB. Copyright © 2018 Lind et al. 2018 Lind et al. This content is distributed under the terms of the Creative Commons Attribution 4.0 International license.

The nine BGCs that are jointly regulated by BrlA, AbaA, and WetA include the BGCs for ferricrocin, DHN-melanin, fumigaclavines, endocrocin, gliotoxin, fumiquinazolines, and pyripyropenes, as well as the unknown nonribosomal peptide synthetase (NRPS) cluster 24 and the unknown polyketide synthase (PKS) cluster 31 (Fig. 2). Four of the five known asexual development-specific BGCs (endocrocin, fumigaclavine, fumiquinazoline, and DHN-melanin) are regulated by all three developmental regulators (Fig. 2); the only exception is the conidial PKS BGC for trypacidin, which appears to not be under the control of any of the developmental regulators under the conditions tested. However, the trypacidin pathway-specific transcription factor gene *tpcE* (Afu4g14540) is positively regulated by BrlA (Table S1) (10). While BrlA is required for production of trypacidin when cultures are grown on solid plates (20), it is possible that the liquid shake conditions used here impact trypacidin production. Aside from these four asexual development-specific BGCs, the other five BGCs that are jointly regulated by all three developmental regulators include the gliotoxin BGC, the intracellular siderophore ferricrocin BGC, the meroterpene pyripyropene BGC, and BGCs 24 and 31 (Fig. 2). Although previous results indicated the involvement of BrlA in regulating gliotoxin biosynthesis (16), it is not yet known whether this mycotoxin is present in asexual spores.

Joint regulation by BrlA, AbaA, and WetA extends beyond the nine BGCs and includes genes involved in sulfur/methionine metabolism (8 genes) and aromatic amino acid metabolism (4 genes) (Table S1), suggesting a connection between primary metabolism (e.g., substrate availability) and secondary metabolism. For example, the GO category cellular amino acid metabolism is significantly enriched in underexpressed genes in the Δ*brlA* and Δ*abaA* mutants (Fig. 1; Table S2). (This category is not significantly enriched in the Δ*wetA* mutant, but 22 of its genes are underexpressed.) This is consistent with previous work linking methionine and tryptophan availability with natural product synthesis (21–23), as well as with evidence that *A. fumigatus* tryptophan metabolism mutants show altered secondary metabolite output (24). Specifically, many NRPS metabolites incorporate aromatic amino acids, such as tryptophan (e.g., fumiquinazoline) or phenylalanine (e.g., gliotoxin), in their carbon skeleton, and gliotoxin itself impacts homeostasis of the methionine cycle (25, 26).

**FIG 3:**
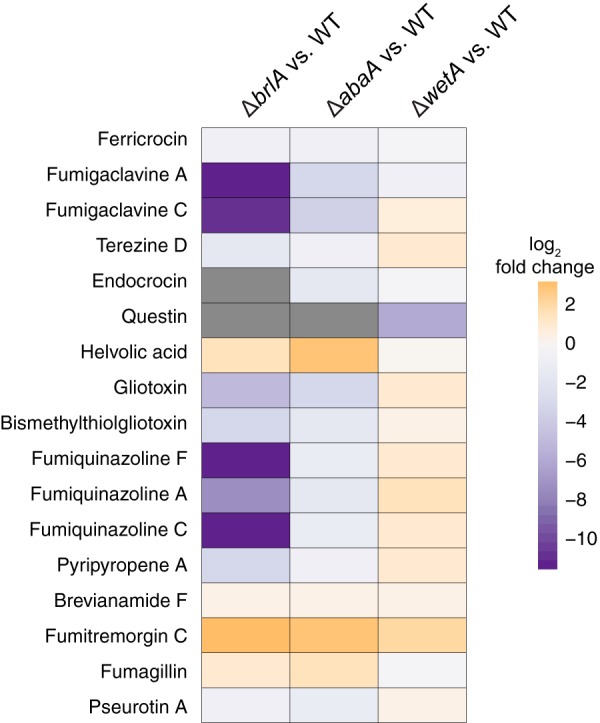
Secondary metabolites are produced at lower levels in the *ΔbrlA* strain relative to the wild type. Summary of metabolite production in *ΔbrlA*, *ΔabaA*, and *ΔwetA* mutants relative to the wild-type strain. Heat map colors represent log_2_ fold change in peak area intensity, and gray indicates no metabolite was detected.

**FIG 4:**
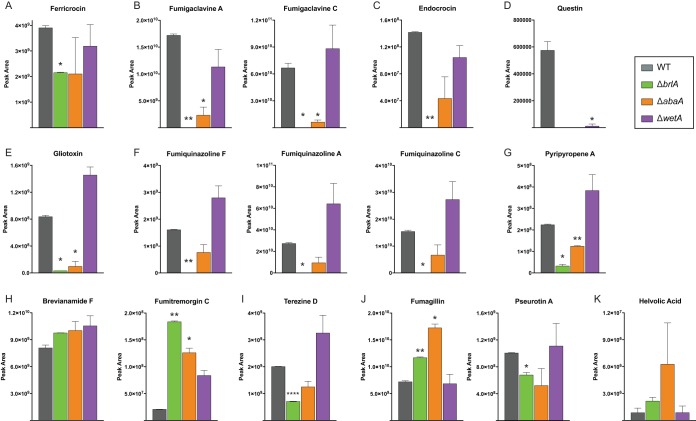
Levels of secondary metabolites produced by wild-type and Δ*brlA*, Δ*abaA*, and Δ*wetA* mutant cultures. Shown is peak area intensity representing total production of representative metabolites from differentially expressed BGCs in *A. fumigatus*. Metabolite analysis was performed using the same two replicate samples from the RNA-seq experiment. Error bars depict standard deviation. Asterisks depict statistical significance compared with the wild type: *, *P* < 0.05; **, *P* < 0.01; and ***, *P* < 0.001.

To examine whether the gene expression changes observed for these BGCs correlate with metabolite production, we performed SM profiling using the same fungal cultures as for the transcriptomic experiments (Fig. 3). In the Δ*brlA* and Δ*abaA* mutant cultures, the metabolite profiles are consistent with the gene expression profiles of their corresponding BGCs. For example, production of ferricrocin, fumigaclavines, endocrocin, gliotoxin, fumiquinazolines, and pyripyropene A is completely abolished or significantly reduced in the Δ*brlA* mutant culture (Fig. 4), mirroring the underexpression of their BGCs in the Δ*brlA* mutant versus WT comparison (Fig. 2). These compounds are also significantly reduced in the Δ*abaA* mutant culture and correlate with the gene expression patterns of their BGCs in the Δ*abaA* mutant versus WT comparison, albeit to a lesser degree than that observed in the Δ*brlA* mutant (Fig. 2 and 4). Ten of the 27 differentially regulated BGCs are under BrlA-specific control (Fig. 2B). Except for the BGC of the extracellular siderophore fusarinine C, the SM products of the remaining nine BrlA-specific BGCs have yet to be characterized. Both BrlA and AbaA regulate the fumitremorgin and fumisoquin gene clusters (Fig. 2B).

One additional gene cluster regulated by both BrlA and AbaA is the fumitremorgin-producing gene cluster. As the *A. fumigatus* Af293 strain used in this study is reported to harbor a point mutation in *ftmD* (Afu8g00200) that renders it incapable of producing the terminal product fumitremorgin C (19), metabolomic analysis on the fumitremorgin BGC was performed using an early pathway precursor, brevianamide F. Total production of brevianamide F is slightly increased in cultures of all three transcriptional regulator mutants (Fig. 4H). Surprisingly, we also detected a significant amount of fumitremorgin C (as determined through *m*/*z* and retention time matched to the fumitremorgin C standard) in the WT strain as well as—at even higher levels—in mutant cultures of all three developmental regulators, suggesting there may be compensation for this mutation or this mutation is absent in our strains (Fig. 4H). FtmD is an *O*-methyltransferase, and it is possible that other *O*-methyltransferases in the genome may function at this step. On the other hand, Kato et al. (19) note that the mutated FtmD enzyme still functions, and it is possible we observed fumitremorgin C production as we grew the fungus under a different condition than the one used in the previous report allowing for FtmD function.

In contrast to the Δ*brlA* and Δ*abaA* mutants, the correlation between Δ*wetA* gene expression and metabolite profiles was much lower. For example, we observed an increase (although not statistically significant) of several SMs, such as the fumigaclavines and endocrocin, in the Δ*wetA* mutant, even though their corresponding BGCs are underexpressed in the Δ*wetA* mutant versus WT comparison (Fig. 2 and (3). Endocrocin is also produced as an early shunt product redundantly by the trypacidin BGC in the strain of *A. fumigatus* used in this study (10) and thus could be attributed to that BGC as well. Although we did not detect the final product trypacidin, minute amounts of the trypacidin precursor question were produced in the wild-type fungus and, to much lesser degree, in the Δ*wetA* mutant (Fig. 4D). The high metabolite production levels in the Δ*wetA* mutant in spite of low gene expression levels could be attributed to the compromised cell wall of this developmental mutant (27), which resulted in increase in SM extraction efficiency compared to other test strains of the fungus.

Assessment of metabolites from both fungal tissue and growth supernatant (secreted) showed that a major fraction of extracted SMs, including all the known conidium-associated SMs (fumigaclavines, endocrocin, fumiquinazolines, and questin), the intracellular siderophore ferricrocin, and pseurotin A, accumulate in the fungal tissue, whereas those of other SMs, such as the fumitremorgins, terezine D, fumagillin, pyripyropene A, and helvolic acid, are secreted into the growth supernatant (see Fig. S1 and Table S4 in the supplemental material). This may be a reflection of the chemical properties of these metabolites and their ability to diffuse or be actively released to the outside of the cell.

10.1128/mSphere.00050-18.1 FIG S1  Semiquantitative RT-PCR results of select genes represented in Fig. 2A. Act1 is the control actin gene, SidC is the NRPS required for ferricrocin synthesis, EncA is the PKS required for endocrocin synthesis, TpcC is the PKS required for trypacidin synthesis, PksP is the PKS required for DHN-melanin synthesis, and 1G17740 is the PKS of cluster 4. The semi-qRT-PCR is in agreement with Fig. 2A, where *sidC*, *encA*, and *pksP* are downregulated (although *sidC* is downregulated to a minor degree) in the Δ*brlA* mutant, whereas *tpcC* and 1G17740 are similar to the wild type—in this case not expressed in either strain. Download FIG S1, PDF file, 0.3 MB. Copyright © 2018 Lind et al. 2018 Lind et al. This content is distributed under the terms of the Creative Commons Attribution 4.0 International license.

10.1128/mSphere.00050-18.2 FIG S2  Concentrations of secondary metabolites produced by wild-type and Δ*brlA*, Δ*abaA*, and Δ*wetA* mutant cultures and located in mycelia and supernatant. Shown are the peak area intensities of representative metabolites from differentially expressed BGCs in *A. fumigatus*. Metabolite analysis was performed using the same two replicate samples from the RNA-seq experiment. Error bars depict standard deviation. Statistics are presented in Table S4. Download FIG S2, PDF file, 1.2 MB. Copyright © 2018 Lind et al. 2018 Lind et al. This content is distributed under the terms of the Creative Commons Attribution 4.0 International license.

Our gene expression data indicate that WetA positively regulates its sole specific target, the iron-coordinating hexadehydroastechrome (HAS) BGC. As metabolite detection of the iron coordination complex of HAS is challenging, we used the monomeric unit of this complex, terezine D, in our metabolite profiling of this BGC. In contrast to the gene expression data, which show that the BGC is underexpressed in the Δ*wetA* mutant versus the WT, we observed that terezine D production is increased in the Δ*wetA* mutant (Fig. 4I). Even though the HAS BGC does not appear to be transcriptionally regulated by BrlA or AbaA, we still observed a decrease in terezine D production in both mutants (Fig. 4I). This could be related to other cellular processes as HAS is a tryptophan-derived metabolite dependent on iron homeostasis (28), with genes in both networks regulated by the BrlA cascade. BrlA and WetA jointly govern the helvolic acid BGC, the fumagillin/pseurotin supercluster, and three unknown BGCs (Fig. 2). Compared to WT levels, production of both fumagillin and helvolic acid is increased in the Δ*brlA* mutant, unchanged in the Δ*wetA* mutant, and substantially increased in the Δ*abaA* mutant (Fig. 4J and (K).

In summary, examination of transcriptional and metabolic profiles of the Δ*brlA*, Δ*abaA*, and Δ*wetA* mutant and WT strains of *A. fumigatus* showed that several BGCs and SMs exhibit BrlA-specific regulation; in contrast, no BGCs or SMs were under AbaA-specific control, and only one showed WetA-specific regulation. Furthermore, several additional BGCs and SMs appeared to be under control of BrlA and WetA or AbaA or under control of all three proteins (Fig. 2). Given that strains lacking *brlA* do not enter asexual development, it is perhaps not surprising that both the gene expression and SM production of asexual development-specific BGCs, such as those for endocrocin and fumigaclavine, are under BrlA control (Fig. 2 and Fig. 4B and C). However, in addition to these spore-associated BGCs and SMs, BrlA also appears to regulate BGCs and SMs, such as helvolic acid and fumisoquin, which are not known to be associated with specialized developmental tissues but rather with vegetative growth, suggesting that BrlA regulation of secondary metabolism extends beyond asexual development. Production of fumisoquin was not assessed due to lack of available purified standard.

### LaeA regulation of secondary metabolism is extensively mediated through BrlA.

**FIG 5:**
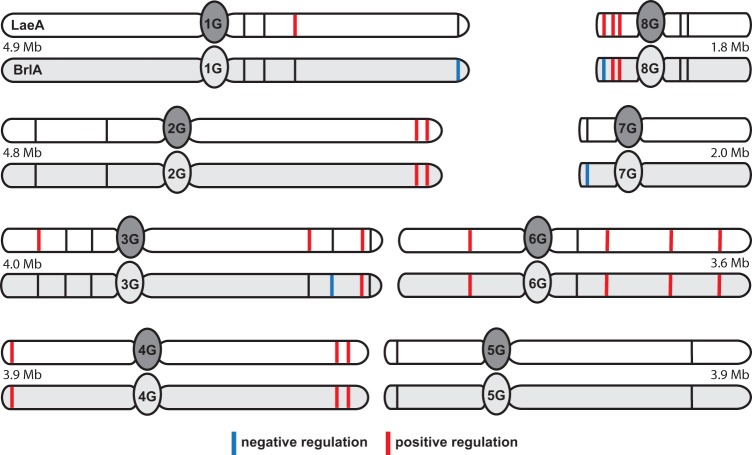
Chromosomal location of all biosynthetic gene clusters involved in secondary metabolism regulated by BrlA and by LaeA. White chromosomes depict BGCs regulated by LaeA, and gray chromosomes depict BGCs regulated by BrlA.

LaeA, a member of the fungus-specific velvet protein complex, is known to regulate secondary metabolism in many agriculturally and medically important filamentous fungi (29). Given the surprising global changes in BGC expression in the *ΔbrlA* mutant as well as the aberrant conidial phenotype previously observed in the *ΔlaeA* mutant (30), we further assessed the genetic relationship between these two global regulators and their governance on secondary metabolism. Global transcriptome comparison between the LaeA and BrlA regulons in *A. fumigatus* shows striking concordance in BGC regulation, with 13/16 of the LaeA-regulated BGCs as determined by microarray-based transcriptome analysis (13) also regulated by BrlA (see Table S5 in the supplemental material). These include the BGCs responsible for the production of DHN-melanin, fumigaclavines, endocrocin, helvolic acid, fumisoquins, gliotoxin, fumiquinazolines, fumitremorgins, fumagillin/pseurotin, and pyripyropenes and three uncharacterized BGCs (cluster 24, an NRPS-based cluster upstream of the gliotoxin cluster, cluster 15, a PKS-based BGC, and cluster 2, a nidulanin-like BGC) (Table S5). Unlike BrlA, which shows both positive and negative regulation of BGCs, LaeA strictly regulates BGCs in a positive manner in *A. fumigatus* at the time point assessed (13) (Fig. 5). Interestingly, except for the fumitremorgin BGC, all of the jointly regulated BGCs are positively regulated by BrlA, further supporting a linked regulatory network between these two global regulators.

10.1128/mSphere.00050-18.7 TABLE S5  Overlap in LaeA- and BrlA-regulated clusters. Download TABLE S5, XLSX file, 0.1 MB. Copyright © 2018 Lind et al. 2018 Lind et al. This content is distributed under the terms of the Creative Commons Attribution 4.0 International license.

**FIG 6:**
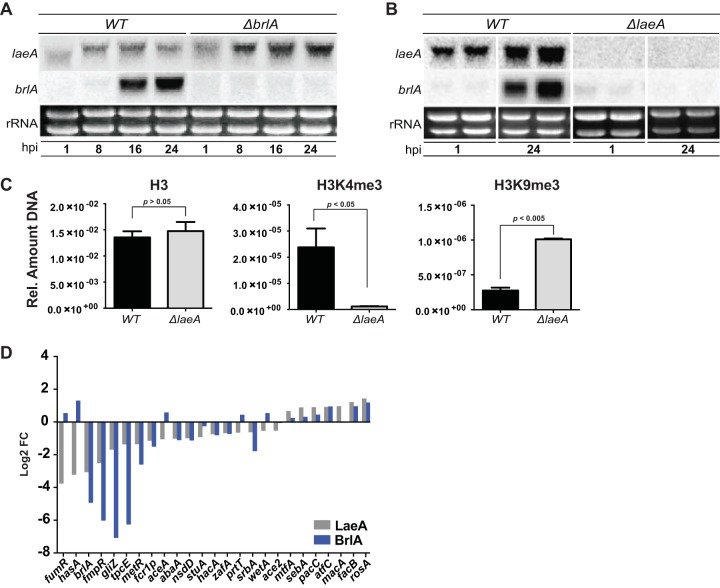
LaeA activity impacts *brlA* transcript levels via chromatin modification. (A) Northern analysis depicting expression of *brlA* and *laeA* in the *ΔbrlA* mutant compared to the WT. (B) Northern analysis depicting expression of *brlA* and *laeA* in the Δ*laeA* mutant compared to the WT. (C) Chromatin immunoprecipitation examining histone modifications of the *brlA* promoter. H3 depicts total histone 3 occupancy, H3K4me3 (histone H3 trimethyl K4) depicts euchromatic marks, and H3K9me3 (histone H3 acetyl K9) depicts heterochromatic marks. Error bars depict standard deviation. Three biological replicates were performed. (D) Predicted transcription factors in *A. fumigatus* with >0.5 log_2_ fold change in the *ΔlaeA* mutant (gray bars). Log_2_ fold changes of the same transcription factors in the *ΔbrlA* mutant are shown by blue bars.

To further decipher the genetic relationship between the LaeA and BrlA transcriptional networks, we assessed the expression of these two genes in each respective deletion mutant. Northern analysis of *brlA* expression and *laeA* expression in the *laeA* and *brlA* deletion mutants showed that, whereas *laeA* expression is not significantly impacted in the Δ*brlA* strain (Fig. 6A), *brlA* expression is significantly reduced in the Δ*laeA* mutant (Fig. 6B), in agreement with previous microarray and RNA-seq data (31, 32). Because LaeA loss is known to be involved in silencing of BGCs through chromatin remodeling (12) and a previous study in *A. nidulans* has shown that LaeA allows for SM expression by counteracting heterochromatin marks on BGC gene promoters, specifically reducing H3K9 methylation through heterochromatin protein 1, HepA (AN1905) (6), we suspected that the regulatory effect of LaeA on *brlA* could be governed through modifications to the chromatin landscape within the *brlA* promoter. Indeed, chromatin immunoprecipitation (ChiP) examining histone modifications of the *brlA* promoter shows that although the histone H3 occupancy at the *brlA* promoter is unchanged between the WT and *ΔlaeA* mutant, there is a substantial decrease of a modification correlating with euchromatin (H3K4me3 [histone H3 trimethyl K4]) in the *ΔlaeA* strain, while the heterochromatic mark H3K9me3 (histone H3 acetyl K9) is greatly enriched (Fig. 6C). Thus, as with BGC regulation, it appears that LaeA epigenetically regulates *brlA* by impeding heterochromatin formation on the *brlA* promoter (Fig. 6). Based on these results, we infer that LaeA regulation of secondary metabolism is significantly mediated through its impact, via chromatin modification, on *brlA* transcript levels.

### Several transcriptional regulators of diverse cellular processes, including the SrbA-regulated hypoxia stress response, are also BrlA and LaeA regulated.

These findings piqued our interest on whether the LaeA-BrlA regulatory relay extends beyond secondary metabolism. To address this question, we compared the differential expression profiles of all *A. fumigatus* transcription factors (TFs) in both the Δ*laeA* mutant versus WT comparison and in the Δ*brlA* mutant versus WT comparison. We found that, similar to the observed overlap of differential expression profiles for BGCs (Table S5), many LaeA-regulated TFs are also regulated by BrlA (Fig. 6D). A detailed assessment of functionally characterized TFs showed that both LaeA and BrlA positively regulate TFs found within BGCs (GliZ [Afu6g09630], FmpR/FapR [Afu6g03430], TpcE [Afu4G14550], and FsqA [Afu03430]) as well as a series of TFs involved in sulfur metabolism (MetR [Afu6g07530]), sexual development (NsdD [Afu3g13870]), asexual development (StuA [Afu2g07900], and AbaA [Afu1g04830]), the unfolded protein response (HacA [Afu3g04070]), zinc response (ZafA [Afu1g10080]), and the hypoxia response (SrbA [Afu2g01260]) (Fig. 6D). In addition, we also observe that both LaeA and BrlA negatively regulate a series of TFs involved in fungal morphogenesis (MtfA [Afu6g02690]), virulence (SebA [Afu4g09080]), pH signaling (PacC [Afu3g11970]), acetate utilization (FacB [Afu1g13510]), and repression of sexual development (RosA [Afu4g09710]) (Fig. 6D). It thus appears that a substantial part of the LaeA transcriptional cascade is moderated via BrlA.

To further examine a cellular process independent of secondary metabolism that is regulated by LaeA and BrlA, but not AbaA or WetA, we focused on the SrbA-regulated hypoxia stress response (33). Underexpressed genes in the Δ*brlA* mutant versus WT are enriched for categories associated with stress response and mitochondrial activity, including cellular respiration, mitochondrion, and response to stress (Fig. 1). Among these genes are the hypoxia regulators *srbA* (Afu2g01260) and *srbB* (Afu4g03460) (Table S1). Both transcription factors contribute to virulence and are critical for regulation of iron uptake, heme biosynthesis, and ergosterol synthesis in *A. fumigatus* (33). Previous work has determined that SrbA is a DNA-binding protein that binds upstream of 97 genes in *A. fumigatus* CEA10, 91 of which have orthologs in the Af293 strain used in this study (33). Sixty-nine of 91 (76%) of these genes are underexpressed in the Δ*brlA* mutant versus the WT (Table 2; see Table S6 in the supplemental material). In contrast, the percentages of SrbA-regulated genes were substantially smaller in both the Δ*abaA* mutant versus WT (14/91 genes [15%]) or the Δ*wetA* mutant versus WT (13/91 genes [14%]). Among the genes coregulated by BrlA and SrbA are those in the ergosterol biosynthetic pathway, including the first enzyme in the pathway, Erg1 (Afu5g07780), both 14-α sterol demethylases (Erg11A/Cyp51A [Afu4g06890] and Erg11B/Cyp51B [Afu7g03740]), Erg5 (Afu1g03950), and both C_4_-sterol methyl oxidases (Erg25A [Afu8g02440] and Erg25B [Afu4g04820]) (34, 35). The nitrate assimilation genes *niiA* (Afu1g12840) and *niaD* (Afu1g12830) are also regulated by BrlA and SrbA, linking sporulation and hypoxia to nitrate assimilation, observations noted in earlier studies (17, 36). Examination of the Δ*laeA* transcriptional profile shows near 100% identity of regulation of these genes (13). These findings largely replicate the working model for transcriptional regulation of the hypoxic response previously presented by Chung et al. (33), placing LaeA and BrlA as critical upstream regulators of this pathway.

10.1128/mSphere.00050-18.8 TABLE S6  Shared targets of SrbA and BrlA. Download TABLE S6, XLSX file, 0.1 MB. Copyright © 2018 Lind et al. 2018 Lind et al. This content is distributed under the terms of the Creative Commons Attribution 4.0 International license.

**TABLE 2:** Numbers and percentages of differentially expressed SrbA-bound genes in Δ*brlA*, Δ*abaA*, and Δ*wetA* mutants

SrbA-bound gene group	No. (%) of genes differentially expressed^a^		
	Δ*brlA* mutant in GMM	Δ*abaA* mutant in GMM	Δ*wetA* mutant in GMM
Overexpressed	6 (7)	26 (29)	18 (20)
Underexpressed	69 (76)	14 (15)	13 (14)
No change in expression	16 (18)	51 (56)	60 (66)

a All comparisons are against the wild type.

### A cellular network regulating fungal secondary metabolism as well as diverse cellular processes.

In filamentous fungi, SM production is coupled with the onset of asexual development. In *Aspergillus*, asexual development is governed by the central regulators BrlA, AbaA, and WetA, which are required for the early, middle, and late stages of asexual development, respectively. To investigate how regulation of asexual development is linked to the tissue-specific regulation of secondary metabolism, we examined the global transcriptomic and metabolomic profiles of Δ*brlA*, Δ*abaA*, and Δ*wetA* mutant and WT strains of *A. fumigatus*. We find a distinct role for BrlA in regulating both asexual development-specific and vegetative growth-specific secondary metabolism, as well as diverse cellular processes, including the hypoxia stress response. Interestingly, BrlA’s involvement in SM regulation occurs in the context of the BrlA > AbaA > WetA cascade, whereas the protein’s involvement in the regulation of diverse cellular processes appears to be dissociated from AbaA and WetA. We further find that the BrlA transcriptional program is highly similar to the LaeA transcriptional program and elucidate that LaeA activity impacts *brlA* expression via chromatin modification.

**FIG 7:**
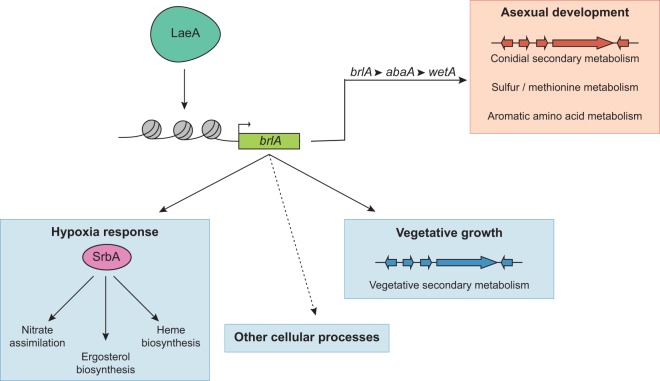
Model framework for the cellular network regulating fungal secondary metabolism and diverse cellular processes. Under our proposed model, the chromatin modifier LaeA, by epigenetically regulating the transcription factor BrlA, controls secondary metabolism in the context of fungal vegetative growth and asexual development, as well as additional cellular processes, such as the hypoxia response.

Interestingly, although LaeA regulation of BrlA had been known since 2007 in *A. fumigatus* (13) and more recently a similar requirement of LaeA for *brlA* expression was noted in *P. oxalicum* (18), no mechanism for this regulation has been uncovered until now. Our results, coupled with those in *P. oxalicum*, suggest that this regulatory relationship is broadly conserved across filamentous fungi, possibly as a mechanism to couple synthesis of appropriate natural products with appropriate developmental stages and allow for a hierarchical framework of LaeA and BrlA function in fungal differentiation processes (Fig. 7).

Both the set of BrlA > AbaA >WetA-regulated genes and the set of BrlA/SrbA-regulated genes show substantial overlap with the set of LaeA-regulated genes (13, 29) (Fig. 6; Tables S4 and S5). Our finding that LaeA epigenetically regulates *brlA* expression provides a mechanistic explanation of these overlaps and alters our understanding of the role of LaeA in the regulation of secondary metabolism in the context of both fungal vegetative growth and asexual development, as well as in the regulation of additional cellular processes. We propose that LaeA, perhaps as a member of the velvet protein complex, regulates key “cellular switches,” with BrlA representing one of these switches (Fig. 7). BrlA is a known transcription factor that was first identified as a regulator of conidiophore development in *A. nidulans* (37, 38). BrlA has also been characterized in several *Aspergillus* and *Penicillium* species, and its regulatory functions have always been associated with sporulation and frequently with secondary metabolism (16, 39–41). The regulatory elements in the *brlA* enhancer have been extensively characterized and are thought to include transcriptional complexes comprised of several regulatory proteins, including two velvet protein family proteins, VosA and VelB (reviewed in reference 39). It is possible that LaeA associates with one or more of these proposed positive-acting transcriptional complexes to inhibit heterochromatic marks on the *brlA* promoter and allow for its activation.

The global nature of BGC regulation by BrlA was surprising and accounts for the majority of LaeA-regulated SMs. Interestingly, of the nine characterized BGCs that our results suggest are identically regulated by both proteins, six do not contain a pathway-specific transcription factor, and of the other three, the BGC-specific transcription factors (TpcE [Afu4g14540], FsqA [Afu6g03430], and GliZ [Afu6g09630] [Table S1]) are highly regulated by BrlA. Thus, it appears that, minimally, these nine BGCs are induced by LaeA-mediated BrlA activation. However, not all BGCs were similarly regulated by LaeA and BrlA, suggesting that they may require LaeA activation through other or additional “cellular switches,” that they may be solely (positively or negatively) regulated by BrlA, or that they may be regulated through LaeA- and BrlA-independent cascades. Since both LaeA and BrlA are present in other fungal genera, including *Penicillium* and *Talaromyces*, and the secondary metabolites produced by organisms in these genera are distinct from those produced by *A. fumigatus*, it will be of future interest to address how conserved global molecular circuitries are rewired to control species-specific processes such as secondary metabolism (42).

Finally, our work shows that BrlA is the likely mediator of many of the known LaeA cellular cascades, including several associated with *A. fumigatus* virulence, substantially expanding the diversity of cellular processes that appear to be regulated by BrlA. For example, both proteins are critical for activation of members of the aromatic amino acid and sulfur/methionine pathways, which play a role in virulence of this pathogen (43, 44). We also find that BrlA is a key regulator of hypoxia-regulated genes, likely through its regulation of SrbA and SrbB, the two key transcription factors critical for hypoxia adaptation in *A. fumigatus* (33, 45). SrbA is also important in azole resistance through its regulation of the ergosterol biosynthetic pathway (46), and our work uncovers a direct signaling pathway from LaeA to BrlA to SrbA/B to ergosterol gene expression which may reveal new avenues to study the expanding threat of antifungal resistance in *Aspergillus* species (47).

## MATERIALS AND METHODS

### Fungal strains and growth conditions.

All strains used in this study are listed in Table 3. The three developmental mutant strains studied (Δ*brlA*, Δ*abaA*, and Δ*wetA*) are deficient in asexual reproduction to various degrees. While both the Δ*abaA* and Δ*wetA* mutants produce conidiophores and spores, their structures are aberrant; the Δ*brlA* mutant does not produce conidiophores or spores (27, 48). Fungal strains are maintained in −80°C glycerol stocks and activated on glucose minimal medium (GMM) at 37°C (49). For RNA-seq analysis, 2.5 × 10^6^ spores of the WT, five plates of the *ΔbrlA* mutant (with fungal hyphae point inoculated onto each plate and allowed to grow to the extent of the dish), two plates of the *ΔabaA* mutant (with fungal hyphae point inoculated onto each plate and allowed to grow to the extent of dish), and 1 × 10^7^ spores of the *ΔwetA* mutant were inoculated into 500 ml of liquid YPD (1% yeast extract, 2% peptone, 2% glucose) and grown under a 250-rpm shaking condition for 24 h at 37°C to synchronize development between strains. The size of the inoculum chosen was previously determined to provide comparable fungal mass after 24 h of incubation under the above condition. In a sterile environment, fungal mycelia were filtered through Miracloth (EMD Millipore) and thoroughly washed in phosphate-buffered saline (PBS) to remove residual YPD. Equal amounts of mycelia were transferred into three flasks containing 250 ml of liquid GMM and incubated at 30°C under a 250-rpm shaking condition to induce development and conidiation (30). Approximately equal amounts of mycelia were removed from all fungal strains at 48 h postinduction, flash frozen in liquid nitrogen, lyophilized, and stored at −80°C until used for RNA extraction and downstream RNA sequencing. For metabolomics analysis, all fungal cultures were left to incubate, and total cultures (growth supernatant and fungal mycelia) were harvested at 96 h postinduction and then frozen at −80°C until ready to be extracted and analyzed.

**TABLE 3:** Strains used in this study

Strain	Identifier	Genotype	Reference
Af293	WT	Wild type	63
TJW54.1	*ΔlaeA* mutant	*ΔlaeA*::*A. parasiticus pyrG*;* pyrG1*	30
ΔAf*brlA7*	*ΔbrlA* mutant	*ΔbrlA*::*A. fumigatus pyrG*;* pyrG1*	48
TSGa17	*ΔabaA* mutant	*ΔabaA*::*A. fumigatus pyrG*;* pyrG1*	27
TSGw4	*ΔwetA*	*ΔwetA*::*A. fumigatus pyrG*;* pyrG1*	27

### RNA isolation and sequencing.

Total RNA was extracted with QIAzol reagent (Qiagen) from freeze-dried mycelia harvested at 48 h postinduction of asexual development following the manufacturer’s protocol and further purified using silica membrane spin columns from the RNeasy Plant minikit (Qiagen). Total RNA was subjected to DNase I digestion to further remove genomic DNA contamination. RNA-seq libraries were constructed and sequenced at the Genomic Services Lab of Hudson Alpha (Huntsville, AL) using 50-bp Illumina paired-end-stranded reads. Libraries were constructed with the Illumina TruSeq stranded mRNA library prep kit (Illumina) and sequenced on an Illumina HiSeq 2500 sequencer. Two biological replicates were generated for each strain sequenced, and 28 to 53 million reads were generated for each library.

### Differential gene expression analysis.

Raw RNA-seq reads were trimmed of low-quality reads and adapter sequences using Trimmomatic with the suggested parameters for paired-end read trimming (50). After read trimming, all samples contained between 19 and 49 million read pairs, with the average sample containing 28 million reads. Trimmed reads were aligned to the *A. fumigatus* Af293 version s03_m04_r11 genome from the Aspergillus Genome Database (51, 52). Read alignment was performed with Tophat2 using the reference gene annotation to guide alignment and without attempting to detect novel transcripts (parameter –no-novel-juncs) (53). Reads aligning to each gene were counted using HTSeq-count with the union mode (54). Differential expression was determined using the DESeq2 software (55). Genes were considered differentially expressed if their Benjamini-Hochberg adjusted *P* value was <0.1.

### Functional enrichment analysis.

Functional category enrichment was determined for differentially expressed genes under all conditions tested using the Cytoscape plugin BiNGO (56, 57). To allow for a high-level view of the types of differentially expressed gene sets, the *Aspergillus* GOSlim v1.2 term subset was used (58). The Benjamini-Hochberg multiple testing correction was applied, and functional categories were considered significantly enriched if the adjusted *P* value was <0.05.

### Gene cluster expression.

*A. fumigatus* BGCs were taken from a combination of computationally predicted and experimentally characterized gene clusters involved in secondary metabolism (59, 60). A list of all BGCs used in this study is available in Table S2. BGCs were designated as differentially expressed if half or more of the genes in the BGC were differentially expressed. BGCs were designated as overexpressed if half or more of the genes in the BGC were overexpressed or were designated as underexpressed if half or more of the genes in the BGC were underexpressed. BGCs in which half or more of the genes in the BGC were differentially expressed but did not have half or more genes either overexpressed or underexpressed were designated as mixed expression.

### Semiquantitative RT-PCR analysis.

Semiquantitative RT-PCR analysis was performed using 10 μg RNA, which was digested with DNase I (NEB catalog no. M0303L) to remove any contaminating genomic DNA. cDNA synthesis reactions were performed using the Bio-Rad iScript cDNA synthesis kit (catalog no. 170-8891) according to the manufacturer’s protocols. Fifty nanograms of cDNA was used per reaction to amplify specific fragments using gene-specific primers. The primers used are listed in Table S7 in the supplemental material. Wild-type strain 293 and the Δ*brlA* mutant were grown in liquid shake GMM at 29 and 37°C under conditions similar to those for RNA sequencing.

10.1128/mSphere.00050-18.9 TABLE S7  Primers used for semi-qRT-PCR. Download TABLE S7, XLSX file, 0.1 MB. Copyright © 2018 Lind et al. 2018 Lind et al. This content is distributed under the terms of the Creative Commons Attribution 4.0 International license.

### Metabolomics analysis. (i) Metabolite extraction.

Total cultures (growth supernatant and fungal mycelia) were obtained 96 h postinduction of asexual development. Growth supernatant and fungal mycelia were separated via filtration through Miracloth (EMD Millipore). Prior to extraction of the growth supernatant, residual mycelial debris was pelleted via centrifugation, and 5 ml of the growth supernatant was subjected to solid-phase extraction (SPE) using Evolute ABN SPE columns (Biotage) following the manufacturer’s protocol. The fungal mycelia were washed thoroughly with PBS to remove residual growth supernatant and extracted using ethyl acetate-dichloromethane-methanol (3:2:1 [vol/vol/vol]) with 1% (vol/vol) formic acid (61) coupled with incubation in a water sonicator for 1 h. Both the growth supernatant and fungal mycelial crude extracts were evaporated to dryness using a Thermo Scientific Savant SC250 vacuum concentrator and stored at −20°C until ready for ultrahigh-performance liquid chromatography-mass spectrometry (UHPLC-MS) analysis.

### (ii) UHPLC-MS analysis: equipment overview and analytical methods.

High-resolution UHPLC-MS was performed on a Thermo Scientific Vanquish UHPLC system coupled to a Thermo Scientific Q Exactive hybrid quadrupole Orbitrap MS. The system was operated in both electrospray positive-ionization (ESI^+^) and electrospray negative-ionization (ESI^−^) modes with ion voltages set at 3.5 kV in both modes.

Crude extracts were reconstituted in 0.5 ml of 50% (vol/vol) acetonitrile plus 0.1% (vol/vol) formic acid and syringe filtered through the 0.2-µm-pore polytetrafluoroethylene (PTFE) filter to remove insoluble materials. Ten microliters was injected into the UHPLC-MS system, separated using an Agilent Zorbax Eclipse XDB-C_18_ column (2.1 by 150 mm, 1.8-µm particle diameter), and run using 0.05% formic acid in acetonitrile as the organic phase and 0.05% formic acid in water as the aqueous phase at a flow rate of 0.2 ml/min. The solvent gradient starts at 20% organic for 2 min, followed by a linear increase to 60% organic over 10 min, a linear increase to 100% organic over 1 min, and a final holding at 100% organic for 5 min totaling to 18 min of run time and data collection. The XDB-C_18_ column was equilibrated at 20% organic for 5 min in between each sample injection throughout the sequence.

Purified standards were used to validate compounds analyzed in this study. The standards used were either commercially purchased or kindly given by other investigators as described below: helvolic acid (21580; Cayman Chemical Company), gliotoxin (G9893; Sigma-Aldrich), brevianamide F (HY-100385; MedChem Express), fumagillin (11332; Cayman Chemical Company), pseurotin A (14441; Cayman Chemical Company), fumitremorgin C (11030; Cayman Chemical Company), pyripyropene A (11896; Cayman Chemical Company), terezine D (purified and given by the Schroeder lab at Cornell University), endocrocin and questin (purified and given by the Wang lab at University of Southern California), fumiquinazolines F and A (purified and given by the Walsh lab at Harvard Medical School), trypacidin (purified and given by the Puel lab at the French National Institute for Agricultural Research—Toulouse), and fumigaclavine A (SC-203051; Santa Cruz Biotechnology). Standards for ferricrocin and fumigaclavine C were unavailable, and thus compound abundances were inferred from calculated *m*/*z*.

Data visualization, peak alignment, analysis of full-scan UHPLC-MS data, ion extraction, and metabolite quantitation were performed using Xcalibur (Thermo Scientific) and MAVEN (64). Ionization mode was chosen for each compound based on optimal peak profiles of their respective standards as assessed in both ESI^+^ and ESI^−^ modes. Both total ion chromatograms (TICs) and extracted ion chromatograms (EICs) were generated in GraphPad Prism 7 (GraphPad Software, Inc.) using coordinate data of peak intensity (*y*) versus retention time (*x*) obtained from MAVEN. The area below the peak that corresponds to each compound was used to generate the table for metabolite quantitation in GraphPad Prism 7 (GraphPad Software, Inc.).

### Chromatin immunoprecipitation and real-time qPCR analysis.

Fifty-milliliter cultures of liquid GMM were inoculated with 1 × 10^6^ spores per ml and incubated at 250 rpm and 37°C for 24 h under light. Triplicate cultures were performed for each strain. Chromatin immunoprecipitation was carried out as described previously (62). Antibodies used for ChIP were rabbit polyclonal to histone H3 acetyl K9 (ab10812; Abcam, Inc.), rabbit polyclonal to histone H3 trimethyl K4 (07-473; Upstate), rabbit polyclonal to histone H3 acetyl K9 (ab8898; Abcam, Inc.), and rabbit polyclonal to C-terminus histone H3 antibody (ab1791). Two micrograms of antibody was used per reaction mixture of 200 mg total protein. Amplification and detection of precipitated DNA in real-time qPCR were performed with iQ SYBR Green supermix (Bio-Rad, catalog no. 170-8880) following the manufacturer’s instructions using primers AF brlA(p) F qPCR (CGTACGGGTGTAAGTCTGATC) and AF brlA(p) R qPCR (CTCTGTATCTTCTAGTTCAATGG). Relative amounts of DNA were calculated by dividing the immunoprecipitated DNA by the input DNA. Each PCR was replicated. To normalize the amount of DNA precipitated with histone H3-acetyl K9 and H3-trimethyl K4, the quantities from precipitation with these antibodies were divided by the previously calculated ratio of the anti-C-terminus histone H3 precipitation to input DNA.

### Accession number(s).

All short read sequences are available in the NCBI Sequence Read Archive under BioProject no. PRJNA396210.

### Data availability.

All short read sequences are available in the NCBI Sequence Read Archive. Differential gene expression from RNA-seq analysis is presented in Table S1, and data from metabolomics experiments are presented in Table S4.
