# Supplementary material for: An LaeA- and BrlA-Dependent Cellular Network Governs Tissue-Specific Secondary Metabolism in the Human Pathogen Aspergillus fumigatus
Source: mSphere. 2018 Mar 14;3(2):e00050-18. doi: 10.1128/mSphere.00050-18 (PMC5853485; doi:10.1128/mSphere.00050-18)
Supplement: FIG S1 [file sph002182495sf1.pdf]

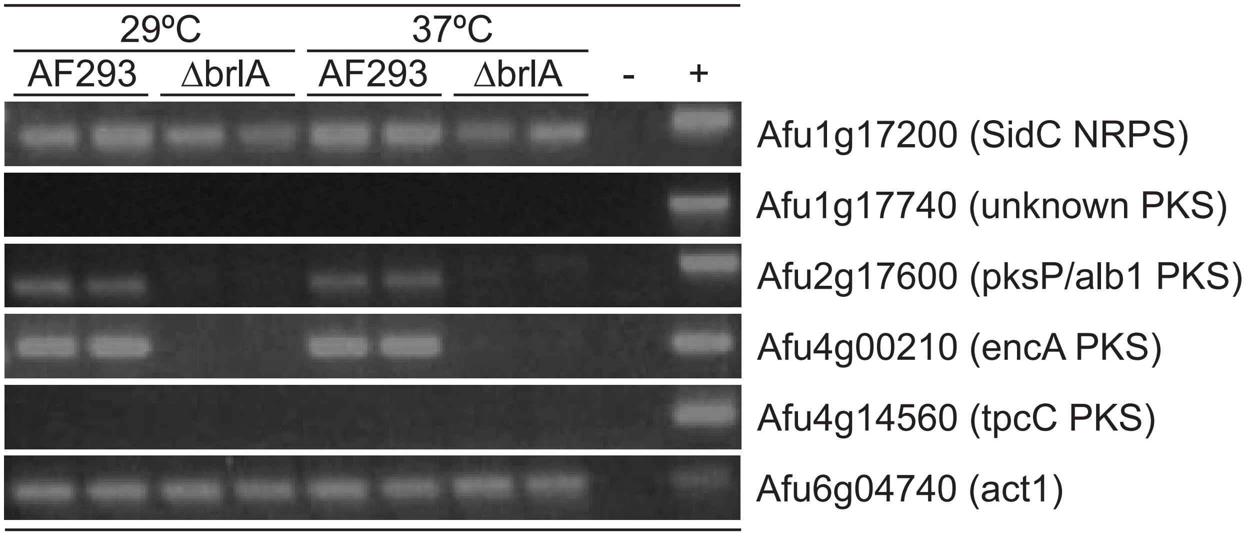
29ºC
37ºC
AF293
ΔbrlA
AF293
ΔbrlA
-
+
Afu1g17200 (SidC NRPS)
Afu1g17740 (unknown PKS)
Afu2g17600 (pksP/alb1 PKS)
Afu4g00210 (encA PKS)
Afu4g14560 (tpcC PKS)
Afu6g04740 (act1)
