# Supplementary figures and images for: An LaeA- and BrlA-Dependent Cellular Network Governs Tissue-Specific Secondary Metabolism in the Human Pathogen Aspergillus fumigatus
Source: mSphere. 2018 Mar 14;3(2):e00050-18. doi: 10.1128/mSphere.00050-18 (PMC5853485; doi:10.1128/mSphere.00050-18)

A

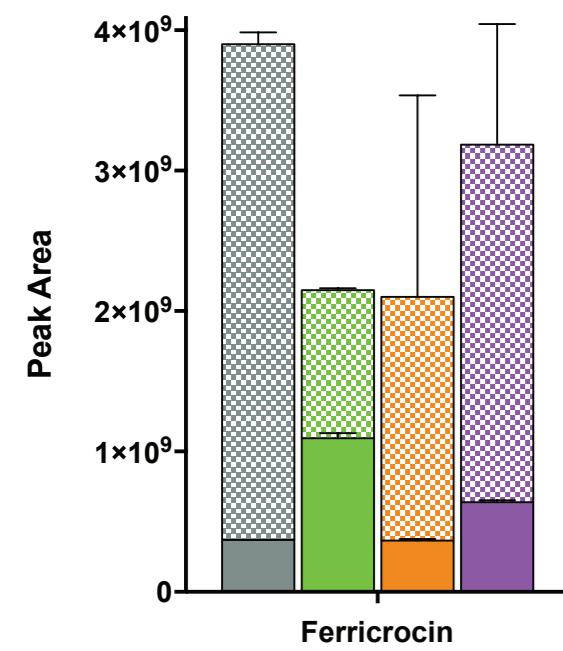

B

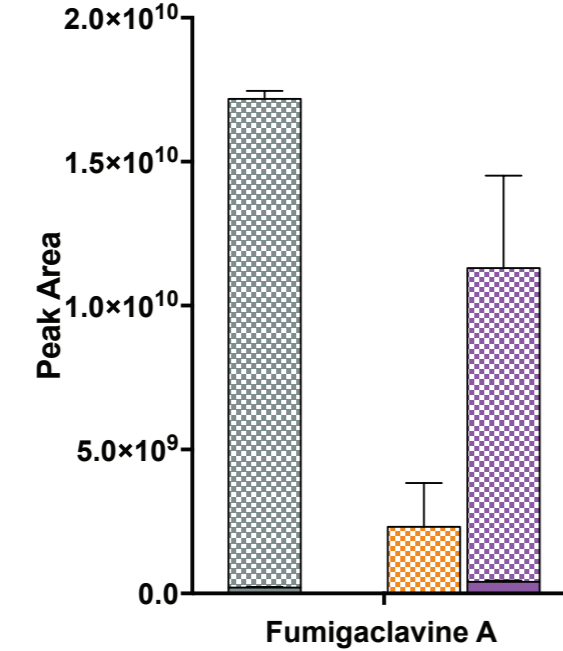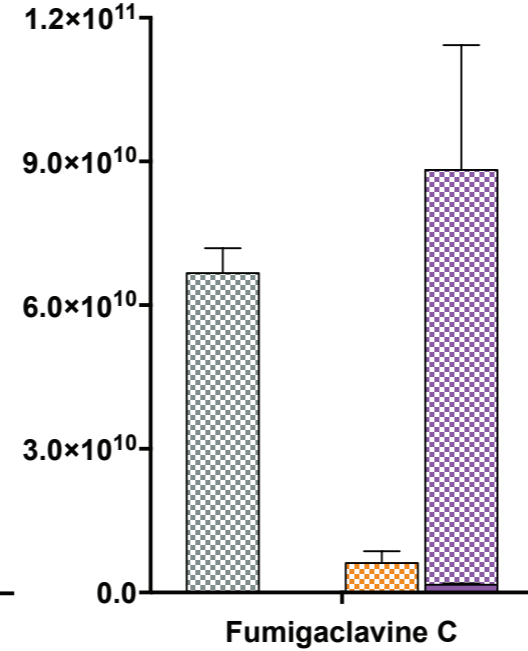

C

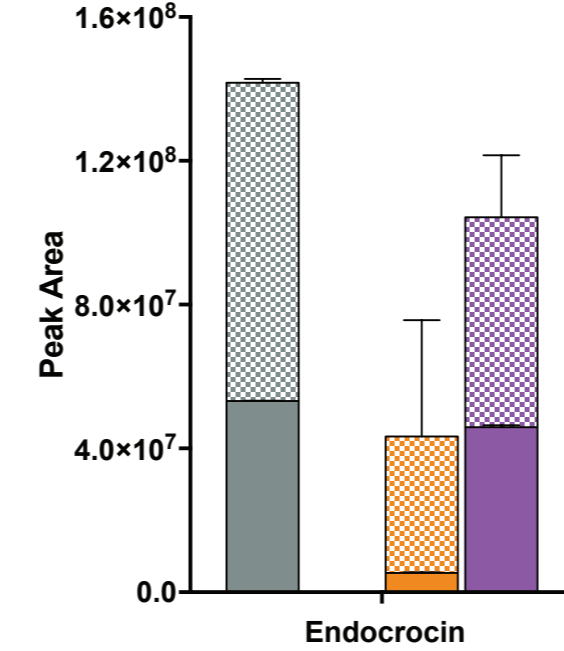

D

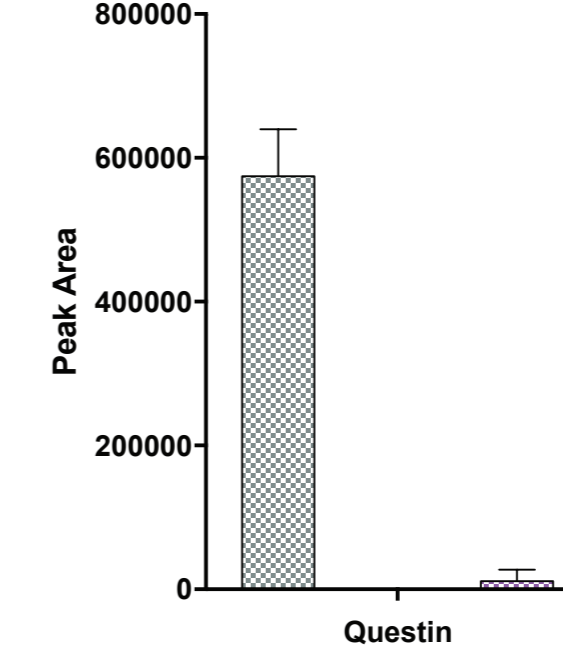

E

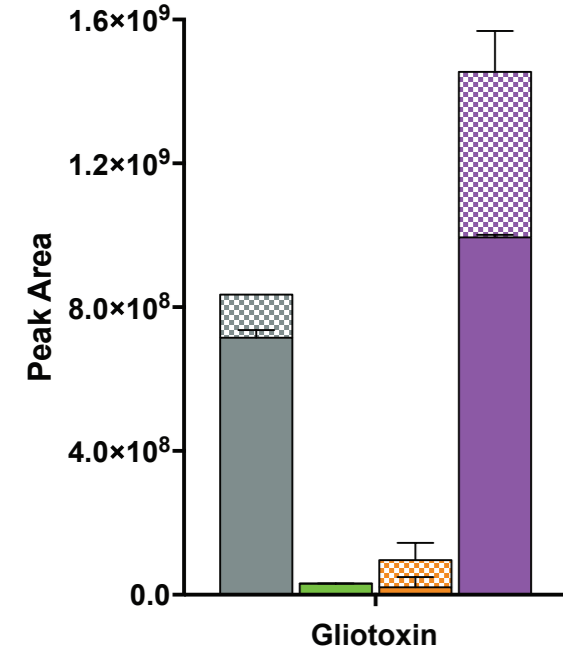

F

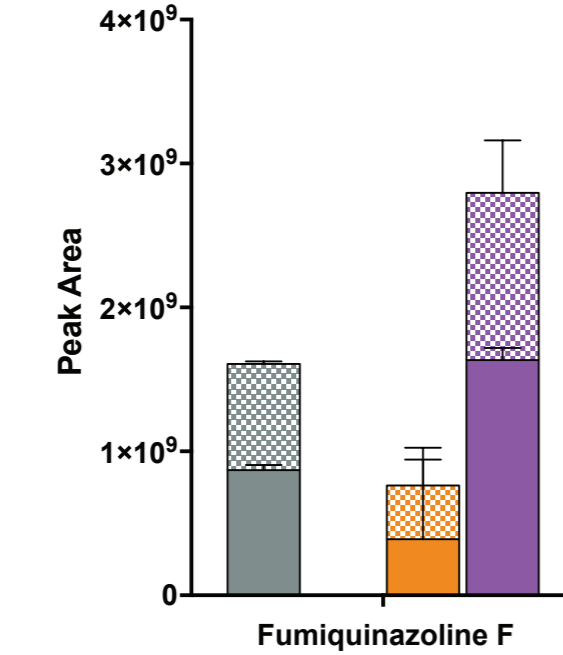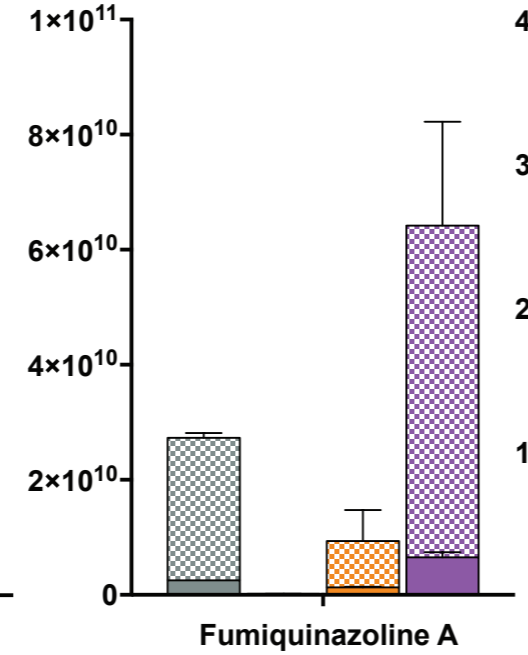

G

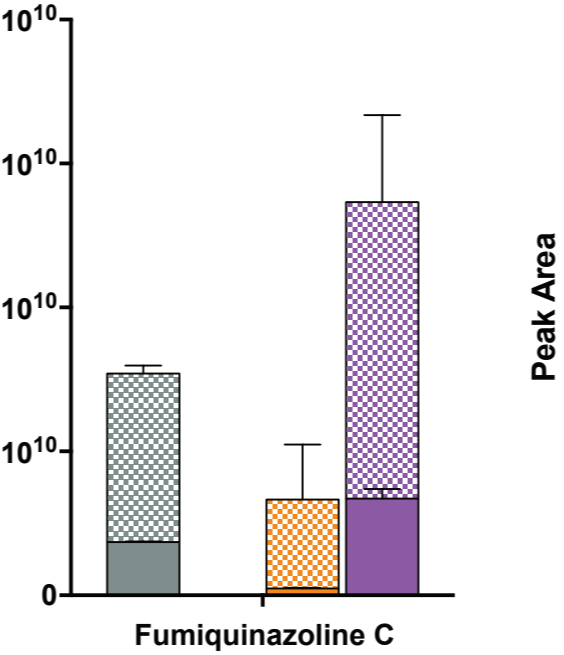

G

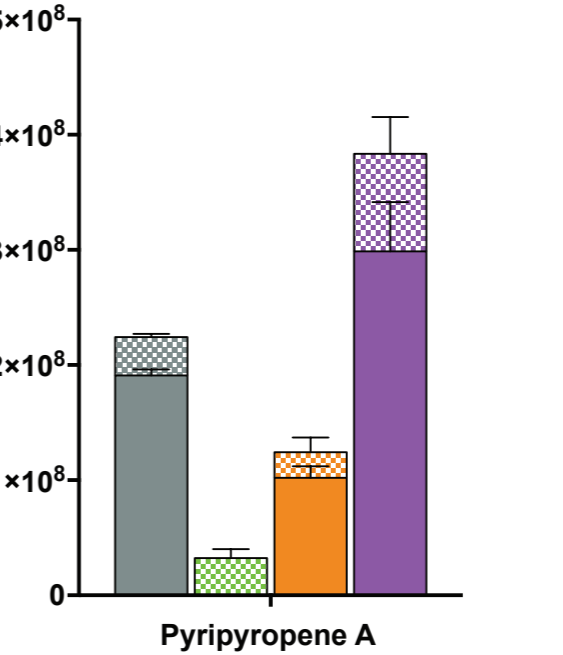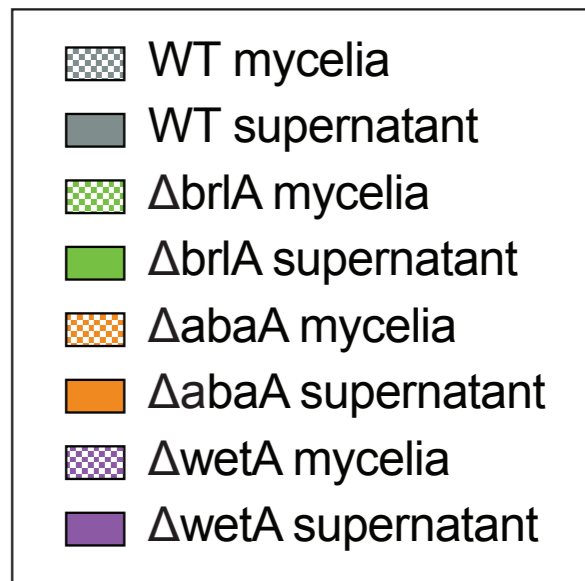

H

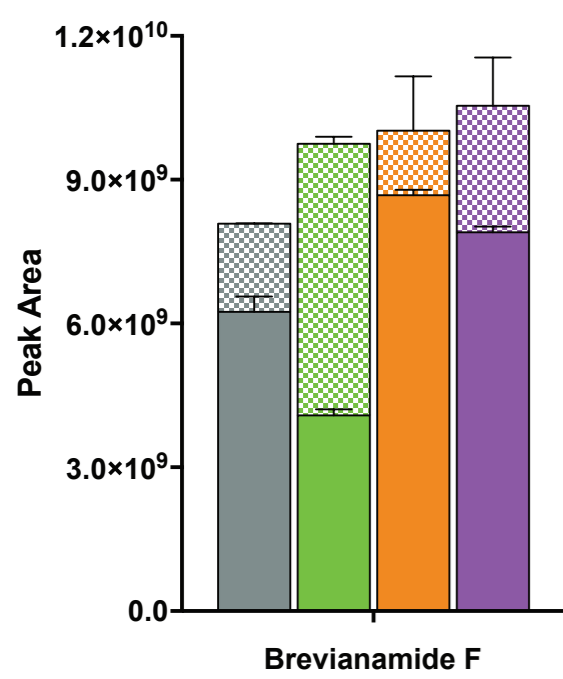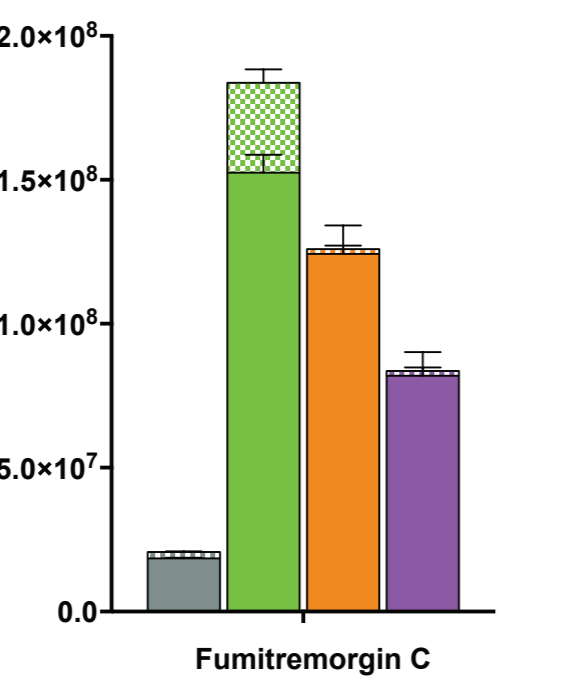

I

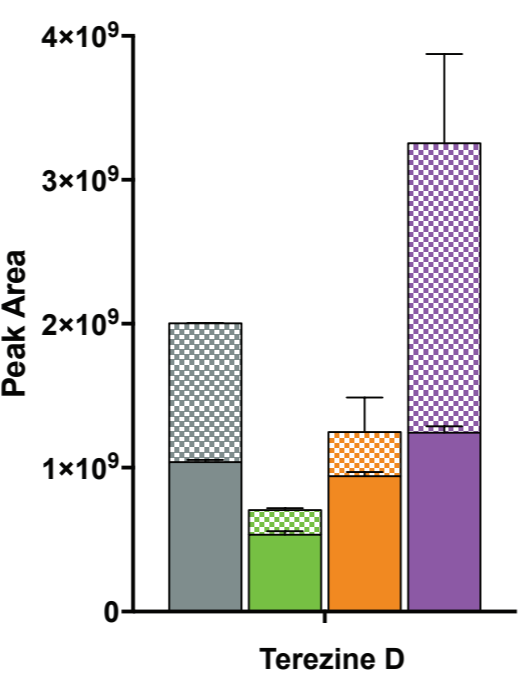

J

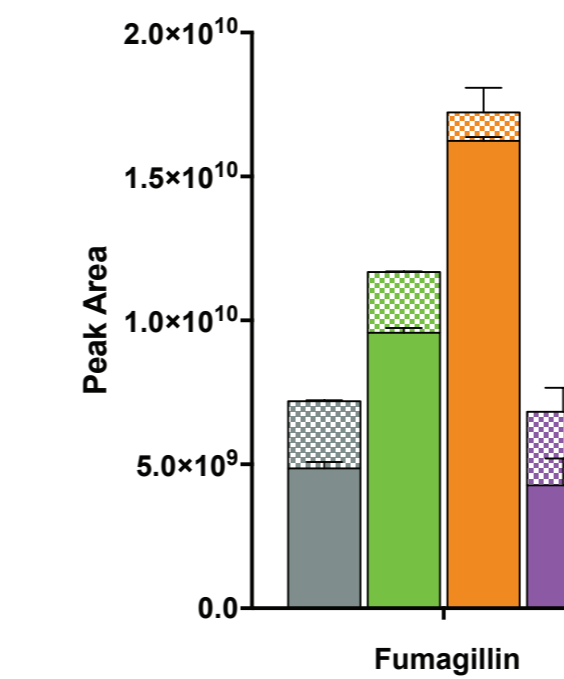

J

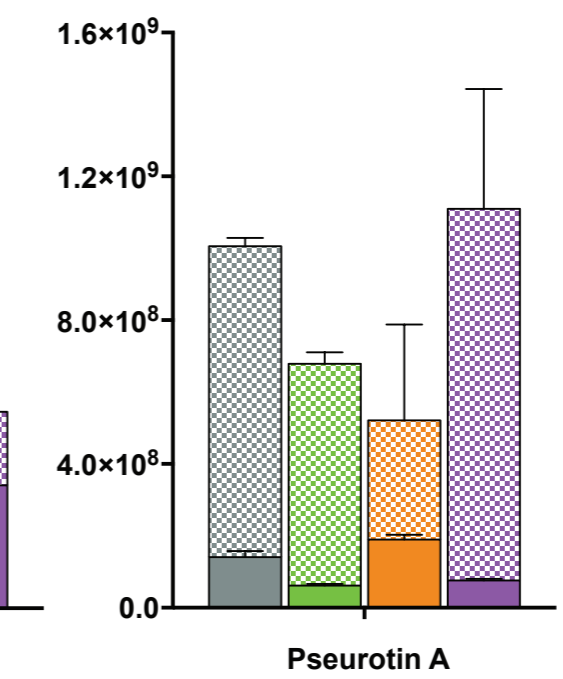

K

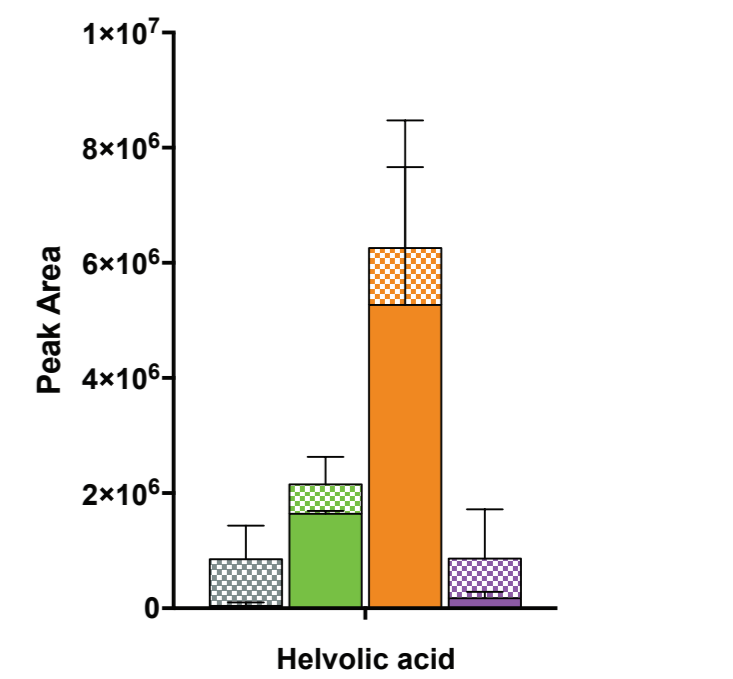

Supplement: FIG S2 [file sph002182495sf2.pdf]
